# Precision Medicine in Relapsed and Refractory Childhood Cancers: Single-center Experience, Literature Review, and Meta-analysis

**DOI:** 10.5041/RMMJ.10342

**Published:** 2018-07-30

**Authors:** Oz Mordechai, Myriam Weyl-Ben-Arush

**Affiliations:** 1 Department of Pediatric Hematology Oncology, Ruth Rappaport Children’s Hospital, Rambam Health Care Campus, Haifa, Israel; 2 Rappaport Faculty of Medicine, Technion–Israel Institute of Technology, Haifa, Israel

**Keywords:** Meta-analysis, NGS, pediatric oncology, precision, solid tumors

## Abstract

**Objective:**

To date, the understanding of pediatric tumor genomics and how these genetic aberrations correlate with clinical outcome is lacking. Here, we report our experience with the next-generation sequencing (NGS) test program and discuss implications for the inclusion of molecular profiling into clinical pediatric oncology trials. We also aimed to explore studies on NGS in pediatric cancers and to quantify the variability of finding actionable mutations and the clinical implications.

**Methods:**

We present a retrospective case series of all patients whose tumor tissue underwent NGS tests during treatment in our department. We also reviewed the literature and carried out a meta-analysis to explore studies on NGS in pediatric cancers.

**Results:**

In 35/37 (94%) patients, we found at least one genomic alteration (GA); mean number of GAs per patient was 2 (range, 0–67), while 164 GAs were detected. Only 3 (8%) patients received precision medicine due to their GAs for a mean of 9 months (range, 5–14 months). Four studies were included in the meta-analysis. The pooled positive actionable mutation rate was 52% (95% CI 39%–66%), and the pooled rate of children who received precision medicine was 10% (95% CI 3%–20%).

**Conclusions:**

In children and young adults with high-risk, recurrent, or refractory malignancies, tumor profiling results have clinical implications, despite barriers to the use of matched precision therapy.

## INTRODUCTION

The annual incidence of childhood cancers is 140–160 *de novo* cases per million children aged 0–14 years. Although survival rates for most childhood cancers have improved in recent decades, pediatric cancer is the leading cause of death by disease in children past infancy,^1^ and the prognosis of children with brain tumors, such as high-grade gliomas, brainstem tumors, and medulloblastomas, as well as metastatic sarcomas and neuroblastomas, continues to be poor.^1^ Prognosis is even more unfavorable with relapse, and standard guidelines for therapies are lacking. The growing ability to analyze the tumors and understand their development and progression opens new options for precision therapies using novel targeted substances. While most tumor cells harbor more than one tumor-propagating change within the different cell-signaling pathways,^2^ progressive or relapsed tumors often display additional molecular changes mediating resistance to standard treatment regimens.^3^

The traditional approach of evidence-based medicine in clinical medicine relies on studies of patient cohorts defined by simple eligibility criteria that demonstrate an average effect of the intervention studied. This is in contrast to the “precision medicine” approach, which is the process of integrating histological and molecular data aiming to find the most suitable treatment for the biological profile of the tumor.^4^

Next-generation sequencing (NGS) tests have advanced rapidly in recent years and include DNA analyses, such as sequencing large numbers of genes (hundreds to thousands) in a single test. These tests can simultaneously detect genomic alterations, such as deletions, insertions, copy number alterations, translocations, and exome-wide base substitutions in all known cancer-related genes.^5^,^6^

Comprehensive DNA sequencing studies have revealed important aspects of the pediatric cancer pathogenesis, and the implementation of precision cancer medicine in pediatric oncology faces unique challenges. Compared with adult cancers, pediatric cancers harbor far fewer genetic alterations.^7^ In general, pediatric cancer is comparatively rare, and most children are cured with conventional therapies. In addition, regulations governing research in children make it difficult to obtain tumor samples for research purposes.^8^ At present, few precision therapies are available to target childhood cancers. Those that are most widely used consist of drugs originally developed for adults.^9^

Ruth Rappaport Children’s Hospital is the main health provider for children with cancer from northern Israel, accepting approximately 120 new pediatric oncology patients per year, while in Israel the average survival rate at 5 years was 80.8% in 2003.^10^ In 2013, our department began to assess the feasibility of integrating precision medicine into the care of children with relapsed and/or refractory cancer. The major tool in our program is the NGS test and trials to provide precision therapy in such cases, with referral to clinical trials or a compassionate use of new drugs. Our institute joined the Innovative Therapies for Children with Cancer program (ITCC) in Europe in 2016 with the aim of coordinating the delivery of new drug trials in our institute.

The understanding of pediatric tumor genomics and how these genetic aberrations correlate with clinical outcome is lacking. We report our experience with our precision therapy program and consider the implications for the effective integration of molecular profiling into clinical pediatric oncology.

## METHODS

We report a retrospective case series of all patients whose tumor tissues underwent NGS tests during treatment in our department. We also reviewed the literature and carried out a meta-analysis to explore studies on NGS in pediatric cancers and quantify the variability of finding actionable mutations and the clinical implications. With the all-important term of “actionability” not uniformly defined, decisions are more often related to differences in the thresholds of evidence used to define “actionability” than to differences between sequencing technologies.^11^

### Case Series

Children and young adults treated in our department since 2013, with relapsed and/or solid tumors, were included in our case series. The pathology formalin-fixed paraffin-embedded (FFPE) slides were sent to Foundation Medicine Lab (CLIA Lab, Boston, MA, USA); DNA was isolated from the FFPE sections cut, and sequencing was performed for exons of 315–406 cancer-related genes and selected introns of ~30 genes to an average depth of >500×. Actionable genomic alterations (GA) were defined as those linked to targeted anti-cancer therapies approved or being evaluated in active registered clinical trials.

### Review and Meta-analysis

In this review, we aimed to explore the literature regarding the finding of actionable (targeted) mutations and, further on, the availability of providing precision treatment to children and young adults with solid cancers. We performed meta-analyses in order to estimate their variability and pooled effects.

The electronic search strategy included the medical literature databases PubMed and Google scholar, using sets of key word combinations: “solid childhood cancers,” “refractory,” or “recurrence”; “pediatric oncology” AND “precision medicine”; “NGS” or “targeted therapy.” All reference lists from the main reports and relevant reviews were searched for additional eligible studies.

Study inclusion criteria were: patients aged >0 and <40 years, with refractory or recurrent solid tumors; type of sample: frozen or paraffin; technically successful tumor profiling tests; NGS tests; reported specified actionable genetic alterations criteria and number of positive results; and reported a targetable therapy following the NGS tests.

The exclusion criteria were age ≥40 years and studies with exclusivity of lymphoproliferative cancers or brain tumors. We choose a cutoff age of 40 years old because the limited sources of studies and the selected studies included both children and young adults.

The outcome variables were:

(Equation 1) $$\%\;Actionable\;genetic\;alterations=\frac{No\;of\;actionable\;alterations}{NGS\;samples}$$

And:

(Equation 2) $$\%\;Precision\;treatments=\frac{No\;of\;patients\;that\;received\;precision\;treatments}{No\;of\;patients\;included\;in\;the\;study}$$

The statistical analysis and graphical presentation were performed using StatsDirect Statistical Analysis Software version 3.1.14 (StatsDirect Ltd, Cambridge, UK).

Heterogeneity of the studies was determined using Cochrane’s *Q* test of heterogeneity. Inconsistency in the study results was assessed by *I*^2^, which describes the percentage of total variation across studies that is due to heterogeneity rather than sample error or by chance. When *I*^2^≥50%, we postulated that there was more than moderate inconsistency. The random effects model was chosen if Cochrane’s *Q* test was *P*<0.1 or *I*^2^≥50%. Otherwise, the fixed effects model was selected. The funnel plot and the Egger test were used to examine publication bias (*P*<0.1 considered as statistically asymmetric funnel plot).

## RESULTS

We collected data on 37 patients (Table 1). Median age at the time of the NGS tests was 11.3 years (range, 0.9–20 years). Malignancies included neuroblastoma (*n*=7), brain tumors (*n*=7), osteosarcoma (*n*=5), rhabdomyosarcoma (*n*=4), Wilms’s tumor (*n*=3), Ewing sarcoma (*n*=3), and others (*n*=8). In 35/37 (94%) patients, we found at least one genomic alteration (GA), and the mean number of GAs per patient was 2 (range, 0–67), while a total of 164 GAs were detected. There were 10.1 variants of unknown significance (VUS) on average, and the reports included an average of 1.5 therapy recommendations per patient (range, 0–14) and an average of 4.8 clinical trials per patient (range, 0–39). Overall, only 3 (8%) patients received precision medicine due to their GAs for a mean of 9 months (range, 5–14 months), while 8 patients received an unspecified biological treatment, such as mTOR inhibitor or multi-kinase inhibitors, for an average of 5.6 months (range, 4–8 months).

**Table 1:** Review of NGS Tests in Our Institute

Patient Age (y)	Diagnosis	Genomic Alterations	Potential Therapies	Potential Clinical Trials	VUS	Precision Therapy (mo)	Biological Therapy (mo)	Germline	Patient Status
10;8	Brain	2	3	4	3	0	0	N/A	DOD
11;3	Brain	5	2	7	6	0	0	Y	AWD
17	Brain	2	4	4	4	8	0	N/A	DOD
15	Brain	5	0	4	5	0	0	N/A	AWD
6;5	Brain	3	2	4	0	14	0	Y	DOD
7;6	Brain	4	2	8	12	0	0	N/A	DOD
8;6	Brain	1	3	2	6	0	0	N/A	AWD
15;2	Colon carcinoma	4	2	11	7	0	0	Suspected	DOD
16;8	Colon carcinoma	67	14	39	52	0	0	Y	AWD
14;11	Ewing	1	0	0	8	0	0	N/A	DOD
18;1	Ewing	4	0	0	3	0	0	N/A	DOD
19;2	Ewing	3	2	4	7	0	0	Suspected	AWD
13	Germ cell	3	8	7	5	0	0	Suspected	NED
3	Hepatoblastoma	1	0	10	8	0	5	N/A	AWD
13;7	Neuroblastoma	3	5	5	15	0	0	N/A	DOD
10	Neuroblastoma	1	0	0	10	0	0	N/A	AWD
9	Neuroblastoma	0	0	0	9	0	0	N/A	DOD
7;8	Neuroblastoma	2	2	8	14	5	0	N	DOD
1;8	Neuroblastoma	1	0	7	4	0	0	N/A	NED
1;4	Neuroblastoma	6	0	0	12	0	0	N/A	AWD
2;7	Neuroblastoma	2	0	4	17	0	0	N/A	DOD
20	Osteosarcoma	8	4	15	9	0	6	Y	DOD
15.3	Osteosarcoma	5	2	6	10	0	0	N/A	DOD
13;3	Osteosarcoma	7	0	8	10	0	5	N/A	DOD
12;9	Osteosarcoma	4	0	0	16	0	0	N/A	DOD
15;5	Osteosarcoma	1	0	0	11	0	0	Suspected	AWD
17;4	Other	1	0	0	10	0	0	N/A	AWD
1;6	Other	1	1	0	9	0	0	N/A	DOD
12;2	Other	0	0	0	12	0	0	N/A	AWD
0;9	Rhabdoid	1	0	6	7	0	0	N/A	NED
12;5	RMS	2	0	4	10	0	4	N/A	DOD
19;6	RMS	3	0	0	13	0	7	N/A	AWD
4;9	RMS	2	0	0	12	0	8	Suspected	DOD
9	RMS	3	2	5	13	0	6	Suspected	DOD
5	Wilms’s	1	0	4	7	0	0	N/A	NED
6;4	Wilms’s	2	0	2	9	0	0	N/A	DOD
6;4	Wilms’s	3	0	2	9	0	4	N/A	DOD

AWD, alive with disease; DOD, dead of disease; mo, months of therapy; N, non-approved; N/A, not available; NED, no evidence of disease; RMS, rhabdomyosarcoma; Suspected, in case of positive family history or suspected mutation; Y, yes, approved.

**Figure 1:**
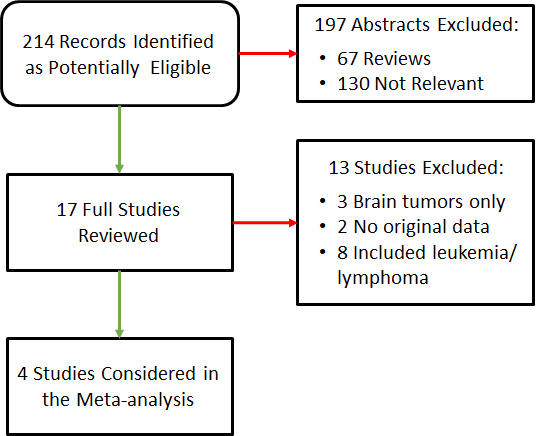
Flow Chart of Studies Included in the Meta-analysis

A total of 214 records were identified as potentially eligible for the meta-analysis, and only four studies were included (Figure 1, Table 2).

**Table 2:** Meta-analysis Sources

Source	Study Name	Median Age (years)	Publication Year	Sample Size	Methods
Worst et al.^12^	The INFORM Pilot Study	13	2016	57	WES, WGS
Harris et al.^8^	iCat Study	13.4	2016	89	Targeted NGS, CGH
Parsons et al.^13^		7.4	2016	121	WES
Harttrampf et al.^14^	MOSCATO-01	10.9	2017	69	Targeted NGS, WES, CGH

CGH, comparative genomic hybridization; NGS, next-generation sequencing; WES, whole-exome sequencing; WGS, whole-genome sequencing.

**Figure 2:**
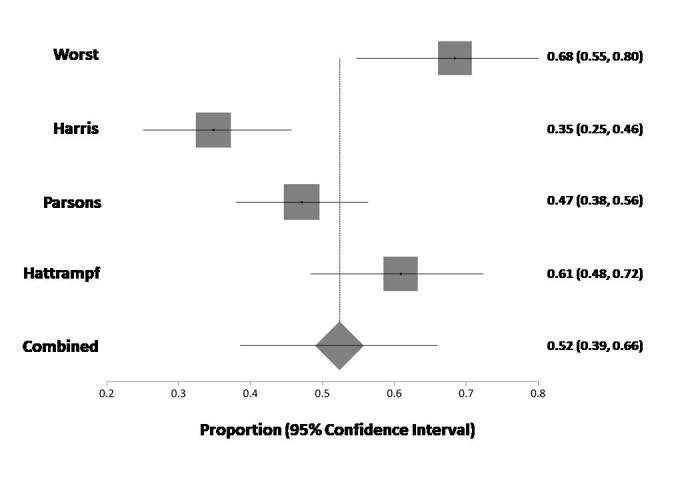
Combined Proportion (Using Random Effect) of % Actionable Alterations in the NGS Tests

The first objective (Figure 2) was to calculate the percent of positive actionable GAs in the total population. Heterogeneity was found (Cochrane *Q*=19.83 [df=3], *P*=0.0002), *I*_2_ (inconsistency)=84.9% (95% CI 50.4%–92.3%). The random effects model was selected, and the pooled positive actionable mutation rate was 52% (95% CI 39%–66%). No publication bias was found.

**Figure 3:**
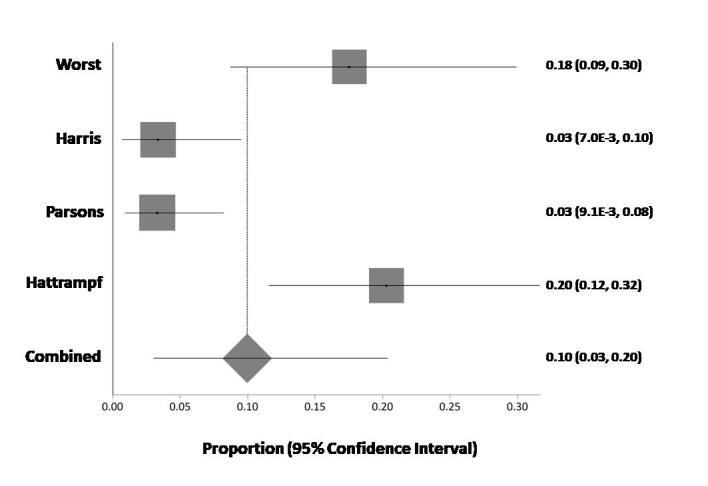
Combined Proportion (Using Random Effect) of % of Patients Who Received Precision Medicine in the Studies

**Figure 4:**
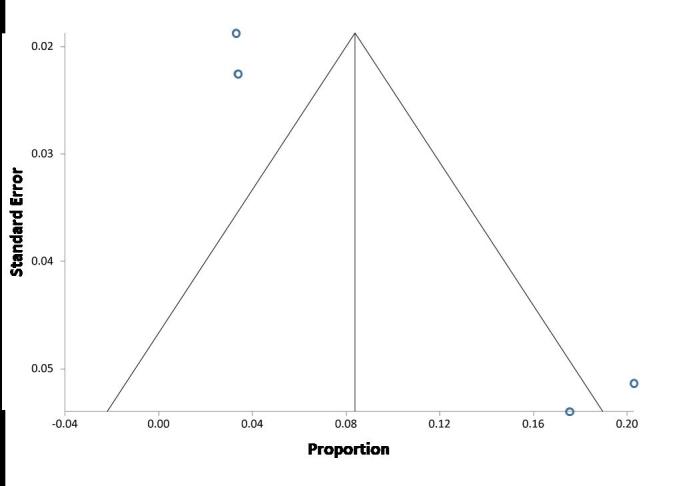
Funnel Plot for the Second Objective: There Is Tendency towards Publication Bias Where More Studies Reported a High Positive Rate of Precision Medicine (Compared to the Pooled Rate)

The second objective (Figure 3) was to calculate the clinical implications of the NGS tests (how many patients received precision medicine) in the total population. Heterogeneity was found (Cochrane *Q*=21.97 [df=3], *P*<0.000), *I*_2_ (inconsistency)= 86.3% (95% CI 58.5%–92.9%). The random effects model was selected, and the pooled precision medicine rate was 10% (95% CI 3%–20%). The funnel plot (Figure 4) suggests a tendency toward publication bias where more studies reported high positive rates of precision medicine (compared to the pooled rate). According to the Egger test, asymmetry was found (*P*=0.021).

## DISCUSSION

In children and young adults with recurrent or refractory solid tumors, tumor-profiling results can have clinical implications, but there are barriers to finding or adapting matched precision therapy. Despite our improved knowledge of genetic alterations in pediatric cancers, precision medicine remains unavailable for the majority of cases. A small number of early-phase pediatric trials include patients whose cancer harbors genetic alterations, including ALK genomic alterations, using ALK inhibitors, and BRAF V6OO mutant tumors, using BRAF or MEK inhibitors.^15^ For example, we published a case report on successful response to precision therapy in a child with aggressive meningioma who was BRAF V6OOE-positive.^16^ But, in our institute, when we suspect a specific driver alteration in a specific disease—such as BRAF fusion alteration in pilocytic astrocytoma^17^—we do not perform a NGS test, but a specific fluorescence *in situ* hybridization test. In adults with lower-grade gliomas, NGS tests are used as a modality for classifying them according to the World Health Organization’s 2016 diagnostic scheme.^18^

In the meta-analysis, potentially actionable alterations were identified in 52% of patients, of which only 10% subsequently received matched therapy, whereas, in our experience, 94% of patients receive NGS reports with potentially actionable alterations and only 8% of them receive precision therapy. We can assume that the high rate of positive tests in our institute is due to our process of finding high pretest probability, as we discuss each case and check genomic databases (such as the Foundation Medicine pediatric database^19^).

These results prove the feasibility of incorporating NGS tests into pediatric oncology practice. Many potentially actionable genetic alterations were detected in a small fraction of patients, meaning that effective pediatric precision oncology therapeutic protocols will require access to a wide range of targeted agents. Precision therapy is not advised if there is a lack of an available matched drug or any inability to enroll in a clinical trial. These results emphasize the need for more clinical trials of targeted therapies in children.

In addition, future efforts will need to relate intra-tumoral heterogeneity, as well as other aspects, such as genetic alterations between primary tumors and relapsed tumors. As technologies improve in the future, one will be able to explore patterns of molecular heterogeneity on the single-cell level to determine how such heterogeneity affects tumor biology and the efficacy of the treatment.^6^

With NGS testing come many ethical questions and concerns, particularly when testing involves germline tissues. In this study, we focused on somatic NGS testing, where there are fewer ethical concerns compared to germline tests. Next-generation sequencing testing may also reveal genetic deviations that are not well understood; these deviations are known as “variants of uncertain significance” (VUS). It can be difficult for families to comprehend that sophisticated NGS tests may actually yield uncertain information, and continued advances in sequencing technologies will, at times, outpace our ability to address the ethical issues surrounding such testing.^20^

There are some study limitations, e.g. heterogeneity of ages and cancer types. There are different types of genetic tests; some of the tests were performed from the initial samples, and some were taken from the recurrence of the disease. Technical difficulties are well known with a high rate of NGS test failures (depending on tumor cell percentages in the specimen, cancer subtype, NGS technique, etc.).

Identification of genetic alterations in childhood cancers with the use of CLIA Lab-certified clinical NGS is feasible. Moreover, finding precision therapies is complicated, and innovative means of bridging this gap are sorely needed.^21^ As shown in our case series, only a small proportion of children with refractory and/or relapsed solid tumors received a precision therapy. From our relevant patients some parents did not want these tests to be performed, some children rapidly deteriorated, and we also had a few tests that technically failed. When we found a driver alteration and a possible treatment, we looked for a relevant clinical trial or a compassionate program.

The goal of contemporary pediatric oncology is to achieve better cure rates with the aid of a comprehensive therapeutic approach that integrates drugs targeting cancer vulnerabilities with inhibitors of processes driven by genomic and/or epigenetic alterations, and also involves immune responses.^2^,^22^ Drug development in pediatric oncology depends on the availability of drugs developed for adults, rather than on the basis of the drug mechanism of action (MoA); therefore, there is limited access to such drugs. For this reason, we joined the Innovative Therapies for Children with Cancer (ITCC) Consortium which chooses a MoA model of drug development, as innovative and rationally designed early-phase trials will promote the development of new drugs for children and adolescents.^23^ Continued drug development, discovery sequencing, and biologic investigation are likely to expand opportunities for precision medicine in pediatric oncology.

## Abbreviations

FFPE: formalin-fixed paraffin-embedded
GA: genomic alteration
ITCC: Innovative Therapies for Children with Cancer
MoA: mechanism of action
NGS: next-generation sequencing
VUS: variants of unknown significance.
